# *Chamaecyparis lawsoniana* Leaf Essential Oil as a Potential Anticancer Agent: Experimental and Computational Studies

**DOI:** 10.3390/plants12132475

**Published:** 2023-06-28

**Authors:** Eman Fikry, Raha Orfali, Samar S. Elbaramawi, Shagufta Perveen, Azza M. El-Shafae, Maher M. El-Domiaty, Nora Tawfeek

**Affiliations:** 1 Department of Pharmacognosy, Faculty of Pharmacy, Zagazig University, Zagazig 44519, Egypt; efhassan@zu.edu.eg (E.F.); azzaelshafae@hotmail.com (A.M.E.-S.); noratawfeek@zu.edu.eg (N.T.); 2 Department of Pharmacognosy, Collage of Pharmacy, King Saud University, Ryiadh 11451, Saudi Arabia; 3 Department of Medicinal Chemistry, Faculty of Pharmacy, Zagazig University, Zagazig 44519, Egypt; sselbaramawy@pharmacy.zu.edu.eg; 4 Department of Chemistry, School of Computer, Mathematical and Natural Sciences, Morgan State University, Baltimore, MD 21251, USA; shagufta.perveen@morgan.edu

**Keywords:** *Chamaecyparis lawsoniana* chemical composition, antioxidant, MTT assay, selectivity index, docking

## Abstract

Cancer remains one of the leading causes of death worldwide, affected by several factors including oxidative stress; and although conventional synthetic medicines have been used to treat cancer, they often result in various side effects. Consequently, there is a growing need for newer, safer and more effective alternatives, such as natural plant products. Essential oils (EOs) are one such alternative, offering a wide range of bioactivities, including antibacterial, antiviral, antioxidant, and anticancer properties. Accordingly, the objective of the present study was to investigate the chemical composition, as well as the antioxidant and anticancer properties of the leaf essential oil of *Chamaecyparis lawsoniana* (CLLEO) belonging to the Cupressaceae family. Totally, 59 constituents were identified by gas chromatography-mass spectrometry (GC-MS) analysis. *cis*-Abienol, *trans*-ferruginol, *α*-cadinol, *δ*-muurolene and *α*-pinene were the major components. The in vitro cytotoxicity study against human breast (MCF-7), colon (HCT-116), lung (A-549), hepatocellular (HepG-2) carcinoma cells using MTT assay indicated a promising cytotoxic activity against all the tested cancer cells, particularly HepG-2, with significant selectivity indices. CLLEO exhibited weak antioxidant activity according to the DPPH, ABTS and FRAP assays. In silico docking of these constituents against the epidermal growth factor receptor (EGFR), the myeloid cell leukemia-1 (Mcl-1) and caspase-8 using Molecular Operating Environment (MOE) software demonstrated good binding affinities of the components with the active site of these targets. These findings suggested using CLLEO, or its individual components, as a potentially viable therapeutic option for managing cancerous conditions.

## 1. Introduction

Last decades, medicinal and aromatic plants played fundamental roles in the area of therapeutics worldwide [1]. Among several secondary metabolites produced from such plants, essential oils (EOs) gained a great interest owing to their complex chemical framework besides their efficacy in several traditional healing approaches [2]. The biological activities of EOs as well as their possible mechanisms of action and pharmacological targets have been reported by several preclinical studies demonstrating their antimicrobial, anticancer, anti-inflammatory, antioxidant, antidiabetic, and spasmolytic activities in several in vitro and in vivo models [2,3,4,5,6].

Recently, species of the family Cupressaceae are considered as a valuable source for various bioactive natural products including EOs [7,8,9,10,11,12]. Various studies have explored the cytotoxic properties of EOs from different species in this family, revealing their potential as effective drugs for treating cancer [12,13,14,15,16,17,18,19]. *Chamaecyparis lawsoniana* (A. Murray) Parl. (Syn. *Cupressus lawsoniana* A. Murray) is a member of the family Cupressaceae which is also named ginger-pine, Lawson’s cypress or Port-Orford-Cedar. It is a huge tree with 43–55 m height that is native to North America with a limited distribution to the coastal forests of Northern California and Southwestern Oregon in the USA [20,21]. Regarding the chemical composition of its EO, few reports from Belgium [22], Iran [23], Spain [21], Greece [24], and Belgrade [25] have revealed the chemical constitution of *C. lawsoniana* EOs. In addition, the literature survey indicated that only one report has concerned with its biological activities revealing its antibacterial and antifungal effects [25,26,27]. Nevertheless, there are no reports about the other possible biological activities including antioxidant capacity and cytotoxic potential of CLLEO.

Cancer is one of the most serious diseases around the world and is exponentially increasing with recently recognized lifestyles. Globally, it was recorded as the primary cause of death since in 2020 about 19.3 million new cancer cases were reported besides 9.9 million cancer fatalities [28]. One of the principal issues with cancer cells is their capability to evade apoptosis via unidentified mutations, leading to accumulation of cells, that in consequence migrate to several body parts [29]. Therefore, drugs which affect cancer cells without influencing normal cells, through restoring the apoptosis mechanisms in the cancer ones and that able to avoid multidrug resistance are considered as effective anticancer drugs [30]. Moreover, the toxicity of the existing chemotherapeutic agents for substantial limitations in their use. Thus, the innovation of safe drug candidates is considered as an important challenge [31]. In this context, natural products have formed an essential role as anticancer drugs and chemotherapeutic agents for about half a century, e.g., vinblastine, vincristine, paclitaxel, camptothecin and doxorubicin [32]. Hence, the discovery of new natural products with anticancer features has unique concern for medical care purposes.

Oxidative stress emerges from the imbalance between the generated free radicals particularly reactive oxygen species (ROS) and the endogenous antioxidant defense systems. High levels of ROS and peroxides have been revealed to be associated with the pathogenesis of various disorders such as cancer, aging, diabetes, neurodegenerative and cardiovascular diseases [33,34].

With the growing focus on the potential anticancer effects of essential oils (EOs) derived from various plants and herbs, this study aimed to assess the chemical composition of essential oil extracted from *C. lawsoniana* fresh leaves, cultivated in Egypt, using GC-MS analysis. Moreover, it validated its antioxidant capacity and tested its effectiveness on different types of cancer cell lines including human breast (MCF-7), colon (HCT-116), lung (A-549) and hepatocellular (HepG-2) carcinoma cells. Furthermore, investigating the conceivable mechanisms that contribute to its cytotoxic activity was accomplished via in silico molecular docking of the CLLEO main components against different targets included in cancer progression.

## 2. Results

### 2.1. Chemical Composition of CLLEO

**Figure 1:**
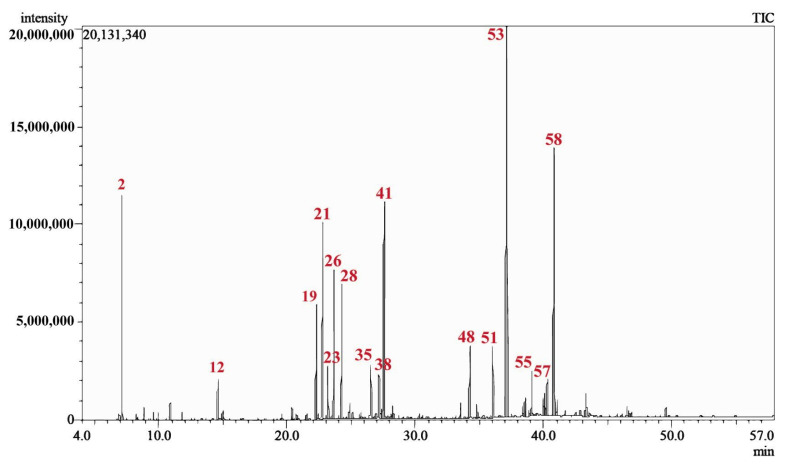
GC/MS chromatogram of *Chamaecyparis lawsoniana* leaf essential oil. Numbers in red are related to Table 1.

**Figure 2:**
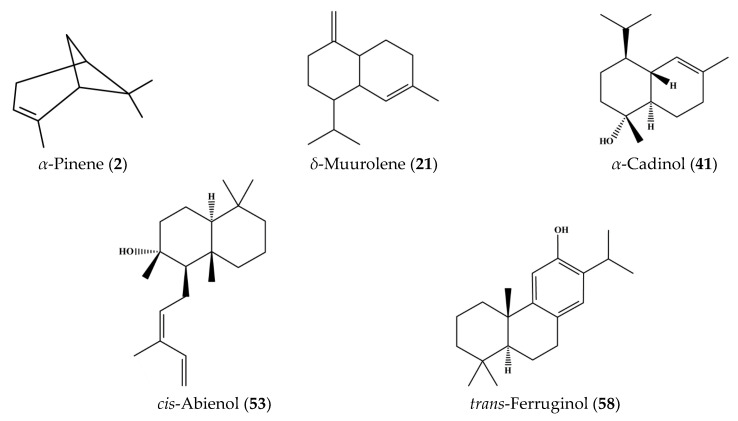
Chemical structures of the main components present in *Chamaecyparis lawsoniana* leaf essential oil.

The hydrodistillation of *C. lawsoniana* fresh leaves resulted in the isolation of a transparent, yellow coloured EO with an average yield of 0.75 ± 0.05% *v*/*w* (from three independent extractions). The GC/MS analysis of CLLEO led to the identification of 59 components, comprising 92.77% of the total composition. The corresponding chemical names of these components and their area percentages are represented in Table 1. Figure 1 displays the GC/MS chromatogram of CLLEO, and the chemical structure of its main constituents are revealed in Figure 2.

### 2.2. Antioxidant Activity

The antioxidant scavenging capacity and reducing antioxidant power of CLLEO were evaluated using DPPH, ABTS and FRAP assays and the results are illustrated in Table 2. In comparison with ascorbic acid, the CLLEO revealed lower radical scavenging potentials against DPPH and ABTS radicals and lower ferric reducing ability, demonstrating relatively weak antioxidant activity.

### 2.3. In Vitro Cytotoxic Potential and Selectivity of CLLEO

**Figure 3:**
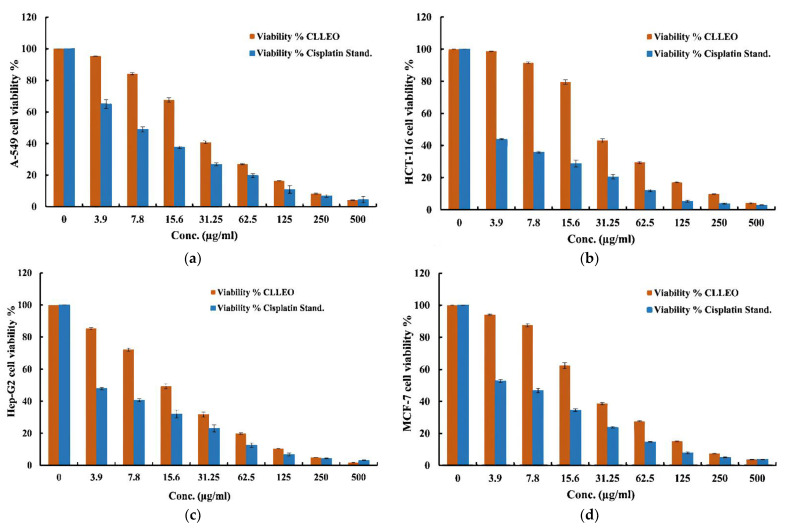
In vitro cytotxicity of *Chamaecyparis lawsoniana* leaf essential oil and cisplatin against: (**a**) human breast (MCF-7), (**b**) colon (HCT-116), (**c**) lung (A-549) and (**d**) hepatocellular (HepG-2) carcinoma cell lines.

In comparison with cisplatin, the cytotoxic effect of CLLEO versus MCF-7, HCT-116, A-549, HepG-2 carcinoma cell lines as well as MRC-5 normal cell line was explored in a concentration dependent manner using MTT assay (Table 3, Figure 3). CLLEO exhibited a pronounced cytotoxic action towards all the tested cancer cell lines. The HepG-2 carcinoma cell line was more sensitive to CLLEO (IC_50_ = 15.34 µg/mL) compared with MCF-7, HCT-116 and A-549 carcinoma cell lines (IC_50_ = 23.74, 28.27 and 25.79 µg/mL, respectively). Moreover, CLLEO revealed a selectivity against the four cell lines (SI > 3) in relative to MRC-5 as shown in Table 3.

### 2.4. In Silico Molecular Docking Study

To gain insights into binding affinity of CLLEO with epidermal growth factor receptor (EGFR) tyrosine kinase (PDB: 1M17) [45], induced myeloid leukemia cell differentiation (Mcl-1) (PDB: 2NLA) [46] and caspase-8 (PDB: 1F9E) [47]; molecular docking was performed.

#### 2.4.1. Docking with Epidermal Growth Factor Receptor (EGFR)

Docking studies of *cis*-abienol, *trans*-ferruginol, *α*-cadinol, *δ*-muurolene and *α*-pinene on epidermal growth factor receptor (EGFR) tyrosine kinase (PDB: 1M17) revealed that the components reached the binding site of the enzyme. Docked components exhibited a good binding affinity as the docking energy score ranged from −5.7955 to −4.9882 Kcal/mol. *cis*-Abienol and *trans*-ferruginol showed H-bond interaction with the acidic Asp831 residue. *trans*-Ferruginol exhibited further stabilization by formation of arene-H interaction with Val702 residue. *α*-Cadinol showed H-bond interaction with Met769 (Table 4).

#### 2.4.2. Docking with Induced Myeloid Leukemia Cell Differentiation (Mcl-1)

Docking studies of *cis*-abienol, *trans*-ferruginol, *α*-cadinol, *δ*-muurolene and *α*-pinene on Mcl-1 (PDB: 2NLA) indicated that the components reached the binding site of the enzyme. Docking energy scores of the docked components ranged from −5.3337 to −4.1318 Kcal/mol. Both *cis*-abienol and *trans*-ferruginol exhibited H-bond interaction with acidic Asp218 residue. *α*-Cadinol showed H-bond interaction with Gly192 residue (Table 5).

#### 2.4.3. Docking with Caspase-8

Docking studies of *cis*-abienol, *trans*-ferruginol, *α*-cadinol, *δ*-muurolene and *α*-pinene on caspase-8 (PDB: 1F9E) exhibited good binding affinity within the binding site of the enzyme as the docking energy scores ranged from −5.4981 to −4.2315 Kcal/mol. *cis*-Abienol showed two H-bond interactions with Asp239 and Tyr244. *trans*-Ferruginol and *α*-cadinol formed H-bond interactions with Cys285 and Asp239, respectively (Table 6).

## 3. Discussion

The essential oil obtained from the fresh leaves of *C. lawsoniana* cultivated in Egypt was subjected, for the first time, to GC/MS analysis to profile its chemical composition. A total of 59 compounds representing 92.77% of the oil were identified. The major identified compound was the bicyclic diterpene alcohol *cis*-abienol (23.43%), followed by the tricyclic diterpene alcohol *trans*-ferruginol (14.31%). Both major compounds belong to the class of oxygenated diterpenes, represented by an overall contribution of 37.74%. Three other components were also detected in resonibally high amounts including the bicyclic sesquiterpene alcohol *α*-cadinol (8.84%), the bicyclic sesquiterpene hydrocarbon *δ*-muurolene (8.57%), and the bicyclic monoterpene hydrocarbon *α*-pinene (6.20%). CLLEO has also been characterized by significant amounts of *γ*-amorphene (4.10%), *δ*-cadinene (3.71%), 6-epi-*β*-cubebene (3.16%), sandaracopimara-8(14),15-diene (2.05%), abietatriene (1.89%), epi-*α*-cadinol (1.74%), pimara-7,15-dien-3-one (1.65%), 1,10-di-epi-cubenol (1.52%), germacrene D (1.32%), *trans*-totarol (1.27%) and terpinen-4-ol (1.20%), while the rest of the identified compounds were found in amounts less than 1%. To our knowledge, there are no studies revealed the chemical composition of the essential oil extracted from *C. lawsoniana* leaves cultivated in Egypt. However, in 1986, Karawya et al. [48] reported that the young twigs essential oil of cultivated specimens from Egypt consisted of limonene (15.8%), gscarene (14.1%), *α*-terpinene (11.2%), and sabinene (10.1%). Moreover, few reports about the chemical composition of *C. lawsoniana* essential oils that were obtained from other sources have been reported. In this regard, De Pooter et al. [22] carried out a study to investigate the chemical composition of *C. lawsoniana* essential oil obtained from leaves cultivated in Belgium. They found that limonene was the main constituent (45–60%) of this essential oil. In a similar work, terpinen-4-ol (22.0%), sabinene (21.0%), camphor (7.8%), citronellol (7.3%), γ-terpinene (7.0%) were identified as dominent components of the aerial parts essential oil extracted from the Iranian *C. lawsoniana* [23]. Additionally, Palá-Paúl et al. [21] reported limonene as the only major component (77.7%) of the essential oils isolated from the young stems and leaves of *C. lawsoniana* cultivated in Spain. Giatropoulos et al. [24] reported that Greek *C. lawsoniana* leaf essential oil composed mainly of 18.5% limonene, 17.1% beyerenne, 15.9% oplopanonyl acetate, and 9.7% methyl myrtenate. More recently, Nikolić et al. analyzed the essential oil obtained from the fresh leaves of *C. lawsoniana* grown in Belgrade [25], demonstrating that the principal constituents of this essential oil were limonene (16.7%), oplopanonyl acetate (14.5%), beyerene (10.1%), and 13-epi-dolabradiene (6.7%). These previous studies demonstrated that the investigated CLLEO had a significant difference in chemical composition and content percentages than that obtained from the same species in different countries. This fluctuation is mainly due to differences in location, climate, harvest period, age of the plant age, distillation method, and type of distillation apparatus used, plant parts used for oil isolation, the environmental conditions and method of analysis [49].

Since a single assay is limited in providing insights into the antioxidant capability of the oil, the CLLEO was subjected to scrutiny using three distinct assays including DPPH, ABTS, and FRAP assays to evaluate its prospective antioxidant activity. The essential oil exhibited weak radical scavenging activity towards DPPH and ABTS radicals as well as low ferric reducing power compared to standard ascorbic acid. These results align with prior research on the essential oil extracted from *Chamaecyparis formosensis* wood, which had a moderate to weak capacity for scavenging DPPH radicals [50]. Moreover, the present findings of the DPPH assay are corroborated by the insignificant anti-DPPH potential exhibited by the essential oil derived from the leaves and fruits of *Chamaecyparis obtuse* [51,52]. Conversely, the essential oils extracted from *C. obtusa* fruits demonstrated a remarkable ability to combat ABTS radicals and a moderate ability to reduce ferric ions [51]. Furthermore, it has been noted that *C. lawsoniana* bark extract demonstrated extraordinary antioxidative properties [53]. The fluctuation in the outcomes could be attributed to the intricate chemical nature of essential oils, which has the potential to yield diverse findings. Consequently, the decreased efficiency of CLLEO could be linked to the lack of phenolic components that contribute to the antioxidative capability [54].

On the other hand, the investigated CLLEO demonstrated a remarked cytotoxic activity against the cancer cell lines under examination. It exhibited a selective potent growth inhibitory activity to HepG-2 cells followed by MCF-7, then A-549 and lastly HCT-116. In addition, it showed a CC_50_ value of 95.17 µg/mL on the normal MRC-5 cells, revealing its selectivity to the carcinoma cell lines, especially towards HepG-2 cell (SI = 6.20). Regarding to the guidelines of the American National Cancer Institute (NCI) that considers IC_50_ values ≤ 30.0 μg/mL as considerably significant for active crude extract or essential oil [55,56], CLLEO could be deemed as a promising anticancer drug candidate. This is considered as the first report about the screening of CLLEO on various cancer cell lines. Regarding to genus *Chamaecyparis*, the review of literature showed that several studies have been conducted to uncover the cytotoxic capabilities of various *Chamaecyparis* species on either the same cancer cells as those used in the current study or on differing cell lines. Remarkably, the findings from these studies were in accordance with the present results. In this sense, *C. obtusa* leaf methanolic extract demonstrated an antiproliferative activity against HCT116 cells at a concentration of 1.25 μg/mL [13]. In addition, the methanolic extract of branches and leaves of *C. obtusa* var. *breviramea* f. crippsii exhibited notable cytotoxic effects against A549, BGC-823, Du145 and MDA-MB-231 with IC_50_ values of 0.94, 1.07, 0.95 and 0.96 μg/mL, respectively. Moreover, various chemical components isolated from the latter species have exhibited cytotoxic effects towards various cell lines. In particular, quercetin demonstrated activity against BGC-823, Hela, and A549 cell lines [14], while 13-epi-toruolsol showed efficacy against BGC-823 and Hela, and 3-epitriptobenzene B displayed activity against BGC-823, Hela, and A549 cancer cell lines [57].

The possible mechanisms involved in the cell death as well as the suggested molecular targets included in the noticed anticancer potential was investigated by accomplishing molecular docking studies for the CLLEO main components (*cis*-abienol, *trans*-ferruginol, *α*-cadinol, *δ*-muurolene and *α*-pinene) using Molecular Operating Environment (MOE) software to explore their binding affinity with the targeted active sites of EGFR, Mcl-1, and caspase-8 proteins and to emphasize its cytotoxic effects on cancer cells.

The epidermal growth factor receptor (EGFR), also named as ErbB1/HER1, is a transmembrane tyrosine kinase receptor protein manifested on the epithelial, mesenchymal, and neurogenic tissues. Upregulation of EGFR is commonly observed in cancer pathogenesis, including metastatic colorectal cancer, non-small-cell lung cancer, head and neck cancer, glioblastoma, breast, and pancreatic cancers. It has a key role in signaling pathways that trigger cell proliferation and apoptosis inhibition. EGFR targeting by anticancer agents suppresses the signal transduction pathways necessary to regulate the growth and proliferation of cancer cells in addition to resistance to cell death [58,59]. The molecular docking of major components of CLLEO on EGFR revealed that the components demonstrated a good binding revealed by the docking scores ranging from −5.7955 to −4.9882 Kcal/mol. Moreover, H-bond interaction with the acidic Asp831 residue was displayed by *cis*-abienol and *trans*-ferruginol. Additional stabilization was shown by *trans*-ferruginol through the formation of arene-H interaction with Val702 residue. *α*-Cadinol, as well, revealed H-bond interaction with Met769.

The programmed cell death, which is known as apoptosis, is a regulatory mechanism that has a key role in the management of cell proliferation in normal physiological processes as well as the pathological conditions. It enhances the removal of unnecessary damaged cells to keep the healthy balance between cell survival and cell death which is missed in case of cancer since the cells be removed are unable to receive the death signals due to a problem in any step of the apoptosis process [60,61]. Therefore, apoptosis could act as a main target for anticancer research. Apoptosis is induced through two main pathways; the intrinsic mitochondrial apoptotic pathway which is initiated by intracellular signals such as DNA damage, oxidative stress, ischemia, radiotherapy, or chemotherapy and triggers the activation of B-cell lymphoma-2 (Bcl-2) family proteins, and the extrinsic death receptor apoptotic pathway which is activated in response to extracellular signals emitted by other cells [60,61,62].

A potent anti-apoptotic protein from Bcl-2 family is the myeloid cell leukemia-1 (Mcl-1) which has a vital role in cellular apoptosis regulation. Mcl-1 overexpression was reported in various types of human cancers and was responsible for the resistance against several anticancer drugs. Additionally, its downregulation was proved to promote the induction of apoptosis and improve the sensitivity toward anticancer drugs [62,63,64]. On the other hand, caspase-8 is one of cysteine proteases involved in the induction of extrinsic apoptosis where it acts as initiator for the apoptosis signal propagation via direct cleavage of downstream effector caspases such as caspase-3. Furthermore, it has a vital role in several cellular processes included in cancer development and progression [65,66]. Consequently, Mcl-1 and Caspase-8 constitute appealing molecular targets for the development of new anticancer drugs. CLLEO main components were docked on Mcl-1 and showed docking energy scores ranged from −5.3337 to −4.1318 Kcal/mol. Both *cis*-abienol and *trans*-ferruginol revealed H-bond interaction with acidic Asp218 residue while *α*-cadinol interacted with Gly192 residue via a H-bond. Moreover, docking of such components on caspase-8 exhibited a good binding affinity within the binding site of the enzyme where the docking energy scores ranged from −5.4981 to −4.2315 Kcal/mol. Two H-bond interactions with Asp239 and Tyr244 were displayed by *cis*-abienol. Also, *trans*-ferruginol and *α*-cadinol created H-bond interactions with Cys285 and Asp239, respectively. Overall, the docked components of CLLEO showed promising binding affinities with the targeted proteins; EGFR, Mcl-1 and caspase-8. The docking results were correlated with the cytotoxic activity of CLLEO, indicating a potential anticancer effect. Therefore, using of each component in this EO, particularly the major ones or the whole EO as anticancer treatment is highly recommended.

In brief, CLLEO could be considered as a promising phytotherapeutic drug candidate in treatment of cancer diseases that acts through the inhibition of cell proliferation and the induction of apoptosis by targeting active sites of the key proteins, EGFR, Mcl-1, and caspase-8. Clinical trials in humans are crucial to guarantee the role of *C. lawsoniana* leaf EO intake as a cancer treatment.

## 4. Material and Methods

### 4.1. Chemicals

The chemicals utilized in this study including 2,2-Diphenyl-1-1-picrylhydrazyl (DPPH), 2,2′-azinobis (3-ethylbenzo-thiazoline-6-sulfonic acid) (ABTS), ascorbic acid, 3-(4,5-dimethythiazol-2-yl)-2,5-diphenyl tetrazolium bromide (MTT), dimethyl sulfoxide (DMSO), and cisplatin, were procured from Sigma (St. Louis, MO, USA).

### 4.2. Plant Material

**Figure 4:**
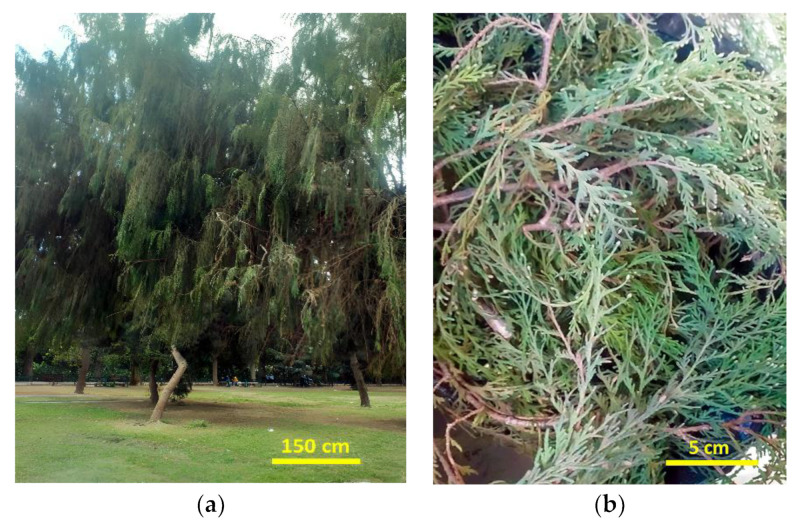
*Chamaecyparis lawsoniana* (A. Murray) Parl. (**a**) Whole tree (**b**) Fresh leaves.

*Chamaecyparis lawsoniana* (A. Murray) Parl. fresh leaves (Figure 4a,b) were gathered in February 2023 from El-Orman Botanical Garden, Giza, Egypt. Taxonomical verification of the plant species was carried out by Eng. Therese Labib, Plant Taxonomy Consultant at the Ministry of Agriculture and Ex-director of El-Orman Botanical Garden, Giza, Egypt. A voucher specimen, with the code ZU-Ph-Cog-0301, was preserved at the Herbarium of the Pharmacognosy Department, Faculty of Pharmacy, Zagazig University.

### 4.3. Essential Oil Extraction

The fresh leaves of *C. lawsoniana* (200 g) were exposed to hydrodistillation by the Clevenger-type apparatus under atmospheric pressure at about 100 °C for 5 h. The obtained essential oil was dried over anhydrous sodium sulphate and kept in brown-colored vials at 4 °C until further chemical and biological analyses. Three extractions were performed, and the average yield was determined.

### 4.4. Gas Chromatography–Mass Spectrometry (GC-MS) Analysis

CLLEO was analysed using Shimadzu GCMS-QP2010 (Kyoto, Japan) fitted with Rtx-5MS fused bonded column (30 m × 0.25 mm i.d. × 0.25 µm film thickness; Restek, PA, USA) equipped with a split–splitless injector. The column temperature was initially kept at 45 °C for 2 min (isothermal) and raised to 300 °C at a rate of 5 °C/min then held at 300 °C for 5 min (isothermal). The injection temperature was 250 °C. Helium carrier gas was used at a flow rate of 1.41 mL/min. The mass spectrometer was scanned over the 35 to 500 *m*/*z* with an ionizing voltage of 70 eV; a filament emission current of 60 mA and an ion source temperature 200 °C. A 1 µL of the diluted sample (1% *v*/*v*) was injected with split mode (split ratio 1:15). The identification of CLLEO components was based on the comparison of their retention indices (RI) and mass spectra (MS) to that reported in Adams library [35], NIST 11 Mass Spectral Library (NIST11/2011/EPA/NIH), Wiley library database 10th edition and the literature data [36,37,38,39,40,41,42,43,44]. The retention indices were assigned in relation to those of a homologous set of standard n-alkanes (C_8_–C_28_) injected under the same conditions. The identified compounds and their percentages are listed in Table 1.

### 4.5. Antioxidant Activity Assays

The antioxidant activity of CLLEO was assessed by three methods with different mechanisms; the 2,2-diphenyl-1-picrylhydrazyl (DPPH) radical scavenging assay according to Elkomy et al. [67], 2,2′-azinobis(3-ethylbenzo-thiazoline-6-sulfonic acid) radical cation (ABTS●+) scavenging assay according to Ling et al. [68] and ferric reducing antioxidant power (FRAP) assay according to Elaasser et al. [69]. The experiments were performed in triplicate and average values were considered. Ascorbic acid was used as a reference compound.

### 4.6. In Vitro Anticancer Assay

#### 4.6.1. Cell Line Propagation

The cytotoxic effects of CLLEO were evaluated in vitro against human breast (MCF-7), colon (HCT-116), lung (A-549), hepatocellular (HepG-2) carcinoma cells and a normal human Lung fibroblast cell line (MRC-5) obtained from the American Type Culture Collection (ATCC, Rockville, MD, USA). RPMI-1640 medium supplemented with 50 µg/mL gentamycin and 10% inactivated fetal calf serum was employed for cells propagation. The cells were kept in a humidified atmosphere with 5% CO_2_ at 37 °C and were sub-cultured two to three times a week.

#### 4.6.2. Cytotoxicity Evaluation Using Viability Assay

MCF-7, HCT-116, A-549, HepG-2 and MRC-5 cell lines were plated into Corning^®^ 96-well tissue culture plates with a cell population density of 5 × 104 cell/well, then incubated for 24 h. A fresh medium containing different concentrations of CLLEO (from 0.25 to 500 µg/mL) was then added. For each 96 well plate, a control was conducted using six vehicle controls containing 0.5% DMSO. The plates were maintained in a humidified incubator with 5% CO_2_ at 37 °C for 24 h. Control cells were incubated without the tested sample. Positive control containing cisplatin was also tested as reference for comparison. After the incubation period, the counts of viable cells were valued by the microplate 3-(4,5-dimethythiazole-2yl)-2,5-diphenyl-tetrazolium bromide (MTT) assay [70]. In brief, the mixture of media and sample was removed and replaced with 100 µL of fresh culture RPMI 1640 medium without phenol red then 10 µL of the 12 mM MTT stock solution (5 mg of MTT in 1 mL of PBS) was added to each well including the untreated controls. After 4 h of incubation, removal of an 85 µL aliquot of the media from the wells was done, and followed by the addition of a 50 µL of DMSO to each well then thoroughly mixed with the pipette and incubated at 37 °C for 10 min. The optical density was then measured at 590 nm with the microplate reader (SunRise, TECAN, Inc., Morrisville, NC, USA) to define the number of viable cells. All experiments were held in triplicate and the percentage of viability was calculated as:

The percentage of viability = (OD_t_/OD_c_) × 100%

where OD_t_ is the mean optical density of wells treated with the tested sample and ODc is the mean optical density of untreated cells. The relation between surviving cells and drug concentration was plotted to obtain the survival curve of each cell line after treatment with the tested sample. The 50% inhibitory concentration (IC_50_, the concentration required to trigger toxic effects in 50% of intact cells), was determined from graphic plots of the dose response curve for each concentration using Graphpad Prism software version 9.4.1 (San Diego, CA, USA). For the normal cell line (MRC-5) it was expressed as CC_50_ (50% cytotoxic concentration).

#### 4.6.3. Statistical Analysis

The results were expressed as the mean values ± standard deviation (SD) of three investigates. Statistica software version 8.0 (StatSoft Inc., Tulsa, OK, USA) was utilized for the statistical analysis. The Tukey’s test was used with a significance level of 5%.

#### 4.6.4. Calculation of Selectivity Index (SI)

To evaluate the efficacy and degree of selectivity of CLLEO, SI value was calculated based on the following formula: SI = [CC_50_ of a normal cell line (MRC-5)/IC_50_ of cancer cell line]. High SI value (>3) suggests a drug with high selectivity against cancer cell lines [71,72].

### 4.7. In Silico Molecular Docking Study

Molecular docking studies of the following components: *cis*-abienol, *trans*-ferruginol, α-cadinol, δ-muurolene and α-pinene were performed to evaluate their binding affinity with the targeted active sites of EGFR, Mcl-1, and caspase-8 proteins. Molecular Operating Environment MOE version 2019.0102 software (Chemical Computing Group, Montreal, Canada) [73] was utilized for the docking studies. Protein and ligand structures were prepared as previously described [74].

The crystal structures of EGFR (PDB: 1M17) [45], Mcl-1 (PDB: 2NLA) [46] and caspase-8 (PDB: 1F9E) [47] were retrieved from the Protein Data Bank (http://www.rcsb.org, accessed on 24 March 2023) [75]. The structures of EGFR, MCl-1 and caspase-8 were prepared through the MOE QuickPrep tool.

*cis-*Abienol, *trans*-ferruginol, *α*-cadinol, *δ*-muurolene, and *α*-pinene were drawn through the Chemdraw^®^ (PerkinElmer Informatics, Inc., Buckinghamshire, UK), then transferred to the MOE using smiles canonical. The energy of the components was minimized with root mean square (RMS) gradient 0.1 kcal/mol and finally preparing a database file.

## Figures and Tables

**Table 1:** Chemical composition of *Chamaecyparis lawsoniana* leaf essential oil.

Peak	Compound Name	Chemical Class	RI_Exp._^a^	RI_Lit_. ^b^	Area%	Identification ^c^
1	*α*-Thujene	Bicyclic monoterpene hydrocarbon	922	924	0.13	MS, RI
2	*α*-Pinene	Bicyclic monoterpene hydrocarbon	931	932	6.20	MS, RI
3	Sabinene	Bicyclic monoterpene hydrocarbon	968	969	0.13	MS, RI
4	*β*-Pinene	Bicyclic monoterpene hydrocarbon	971	974	0.06	MS, RI
5	Myrcene	Acyclic monoterpene hydrocarbon	987	988	0.24	MS, RI
6	*α*-Terpinene	Monocyclic monoterpene hydrocarbon	1012	1014	0.15	MS, RI
7	*p*-Cymene	Aromatic monoterpene hydrocarbon	1020	1020	0.03	MS, RI
8	Limonene	Monocyclic monoterpene hydrocarbon	1024	1024	0.14	MS, RI
9	*γ*-Terpinene	Monocyclic monoterpene hydrocarbon	1055	1054	0.36	MS, RI
10	Terpinolene	Monocyclic monoterpene hydrocarbon	1084	1086	0.15	MS, RI
11	*trans*-Sabinene hydrate	Monocyclic monoterpene alcohol	1117	1098	0.02	MS
12	Terpinen-4-ol	Monocyclic monoterpene alcohol	1176	1174	1.20	MS, RI
13	*α*-Terpineol	Monocyclic monoterpene alcohol	1188	1186	0.21	MS, RI
14	Bornyl acetate	Bicyclic monoterpene ester	1282	1284	0.02	MS, RI
15	*α*-Terpinyl acetate	Monocyclic monoterpene ester	1346	1346	0.12	MS, RI
16	*α*-Ionol	Monocyclic sesquiterpene alcohol	1376	1376	0.30	MS, RI
17	*β*-Elemene	Monocyclic sesquiterpene hydrocarbon	1390	1389	0.12	MS, RI
18	*β*-Caryophyllene	Bicyclic sesquiterpene hydrocarbon	1418	1417	0.15	MS, RI
19	6-epi-*β*-Cubebene	Bicyclic sesquiterpene hydrocarbon	1448	1449	3.16	MS, RI
20	*α*-Humulene	Monocyclic sesquiterpene hydrocarbon	1454	1452	0.14	MS, RI
21	*δ*-Muurolene (*cis*-muurola-4(14)-diene)	Bicyclic sesquiterpene hydrocarbon	1467	1467	8.57	MS, RI
22	*γ*-Muurolene	Bicyclic sesquiterpene hydrocarbon	1477	1478	0.09	MS, RI
23	Germacrene D	Bicyclic sesquiterpene hydrocarbon	1483	1480	1.32	MS, RI
24	*β*-Selinene	Bicyclic sesquiterpene hydrocarbon	1487	1489	0.03	MS, RI
25	*cis*-Cadina-1,4-diene	Bicyclic sesquiterpene hydrocarbon	1494	1495	0.17	MS, RI
26	*γ*-Amorphene	Bicyclic sesquiterpene hydrocarbon	1500	1495	4.10	MS, RI
27	*γ*-Cadinene	Bicyclic sesquiterpene hydrocarbon	1514	1513	0.08	MS, RI
28	*δ*-Cadinene	Bicyclic sesquiterpene hydrocarbon	1524	1522	3.71	MS, RI
29	*α*-Cadinene	Bicyclic sesquiterpene hydrocarbon	1537	1537	0.05	MS, RI
30	*α*-Calacorene	Aromatic bicyclic sesquiterpene hydrocarbon	1543	1544	0.03	MS, RI
31	Elemol	Monocyclic sesquiterpene alcohol	1550	1548	0.59	MS, RI
32	*trans*-Nerolidol	Acyclic sesquiterpene alcohol	1561	1561	0.04	MS, RI
33	Caryophyllene oxide	Bicyclic sesquiterpene oxide	1584	1582	0.19	MS, RI
34	Humulene epoxide II	Monocyclic sesquiterpene epoxide	1611	1608	0.04	MS, RI
35	1,10-di-epi-Cubenol	Bicyclic sesquiterpene alcohol	1617	1618	1.52	MS, RI
36	Junenol	Bicyclic sesquiterpene alcohol	1621	1618	0.04	MS, RI
37	*γ*-Eudesmol	Bicyclic sesquiterpene alcohol	1634	1630	0.14	MS, RI
38	epi-*α*-Cadinol (*tau*-cadinol)	Bicyclic sesquiterpene alcohol	1644	1638	1.74	MS, RI
39	Cubenol	Bicyclic sesquiterpene alcohol	1650	1645	0.21	MS, RI
40	*β*-Eudesmol	Bicyclic sesquiterpene alcohol	1654	1649	0.22	MS, RI
41	*α*-Cadinol	Bicyclic sesquiterpene alcohol	1661	1660	8.84	MS, RI
42	Germacra-4(15),5,10(14)-trien-1-*α*-ol	Monocyclic sesquiterpene alcohol	1690	1685	0.44	MS, RI
43	*cis*-14-nor-Muurol-5-en-4-one	Bicyclic sesquiterpene ketone	1692	1688	0.15	MS, RI
44	6-Isopropenyl-4,8a-dimethyl-1,2,3,5,6,7,8, 8a-octahydronaphthalene-2-ol	Bicyclic sesquiterpene alcohol	1710	1714	0.08	MS, RI
45	7-Hydroxycalamenene	Bicyclic sesquiterpene alcohol	1798	1803	0.07	MS, RI
46	Isopimara-9(11),15-diene	Tricyclic diterpene hydrocarbon	1915	1905	0.10	MS, RI
47	Pimaradiene	Tricyclic diterpene hydrocarbon	1949	1948	0.04	MS, RI
48	Sandaracopimara-8(14),15-diene (13-isopimaradiene)	Tricyclic diterpene hydrocarbon	1969	1968	2.05	MS, RI
49	Kaur-15-ene	Tetracyclic diterpene hydrocarbon	1997	1997	0.43	MS, RI
50	13-epi-Manool oxide	Tricyclic diterpene oxide	2019	2009	0.04	MS, RI
51	Abietatriene	Tricyclic diterpene hydrocarbon	2061	2055	1.89	MS, RI
52	Abietadiene	Tricyclic diterpene hydrocarbon	2087	2087	0.07	MS, RI
53	*cis*-Abienol	Bicyclic diterpene alcohol	2120	2112	23.43	MS, RI
54	Sandaracopimarinal	Tricyclic diterpene aldehyde	2195	2184	0.83	MS
55	Pimara-7,15-dien-3-one	Tricyclic diterpene ketone	2225	2227	1.65	MS, RI
56	Sandaracopimarinol	Tricyclic diterpene alcohol	2282	2269	0.73	MS
57	*trans*-Totarol	Tricyclic diterpene alcohol	2291	2290	1.27	MS, RI
58	*trans*-Ferruginol	Tricyclic diterpene alcohol	2325	2331	14.31	MS, RI
59	*cis*-Ferruginol	Tricyclic diterpene alcohol	2337	2340	0.44	MS, RI
	Total identified				92.77	
	Monoterpenes hydrocarbons				7.59	
	Oxygenated monoterpenes				1.57	
	Sesquiterpene hydrocarbons				21.72	
	Oxygenated sesquiterpenes				14.61	
	Diterpene hydrocarbons				4.58	
	Oxygenated diterpenes				42.70	

Compounds are ranked in the order of their elution on Rtx-5MS GC column. ^a^ Retention index defined experimentally on Rtx-5MS column relative to C_8_–C_28_ n-alkanes. ^b^ Reported retention index. ^c^ Identification was established by matching of the mass spectral (MS) data and retention index (RI) values of compounds from Adams library [35], NIST 11 Mass Spectral Library, Wiley Registry of Mass Spectral Data 10th edition, and the literature [36,37,38,39,40,41,42,43,44].

**Table 2:** Antioxidant activity of *Chamaecyparis lawsoniana* leaf essential oil.

	IC_50_ ± SD (µg/mL)		
	DPPH	ABTS	FRAP
CLLEO	116.91 ± 5.73	73.02 ± 4.06	218.64 ± 8.41
Ascorbic acid	10.22 ± 0.56	10.66 ± 0.89	20.89± 1.25

**Table 3:** Cytotoxicity of *Chamaecyparis lawsoniana* leaf essential oil and cisplatin against some cell lines. CC_50_ (50% cytotoxic concentration) and IC_50_ (50% inhibitory concentration) values are expressed by mean ± standard deviation; SI (selectivity index) = CC_50_ value of normal cell/IC_50_ value of cancer cell.

	MRC-5	MCF-7		HCT-116		A-549		HepG-2	
	CC_50_	IC_50_	SI	IC_50_	SI	IC_50_	SI	IC_50_	SI
CLLEO	95.17 ± 3.71	23.74 ± 1.72	4.01	28.27 ± 2.13	3.37	25.79 ± 1.95	3.69	15.34 ± 0.96	6.20
Cisplatin	19.43 ± 3.66	5.69 ± 0.37	3.41	2.51 ± 0.67	7.74	7.51 ± 0.82	2.59	3.68 ± 0.24	5.28

**Table 4:** Docking details of the selected components of *Chamaecyparis lawsoniana* leaf essential oil on EGFR.

Component	S Score Kcal/mol	3D Protein-Component Interactions
*cis*-Abienol	−5.5184	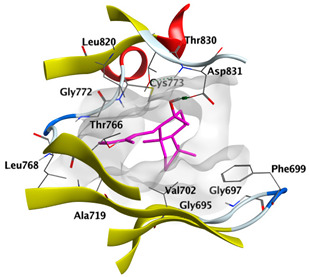
*trans*-Ferruginol	−5.7955	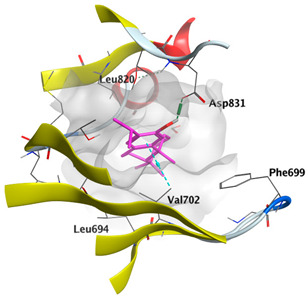
*α*-Cadinol	−5.1630	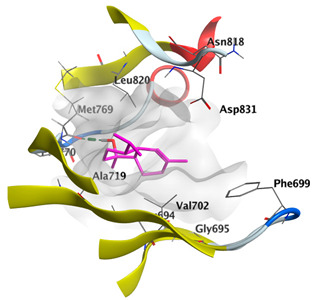
*δ*-Muurolene	−5.7461	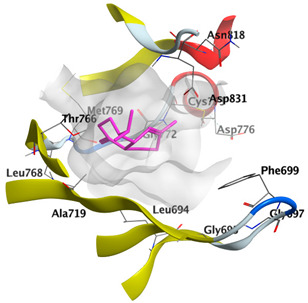
*α*-Pinene	−4.9882	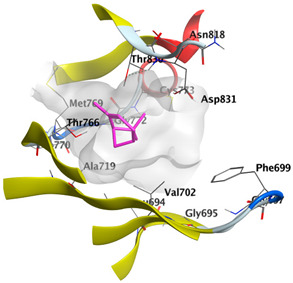

**Table 5:** Docking details of the selected components of *Chamaecyparis lawsoniana* leaf essential oil on Mcl-1.

Component	S Score Kcal/mol	3D Protein-Component Interactions
*cis*-Abienol	−5.3337	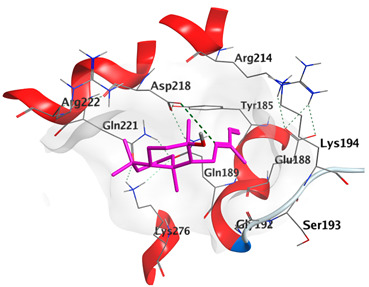
*trans*-Ferruginol	−5.2220	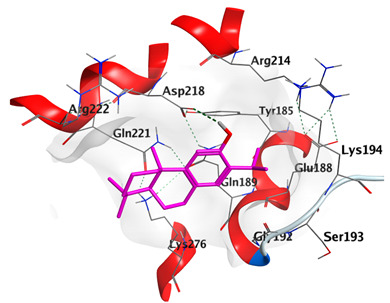
*α*-Cadinol	−4.5189	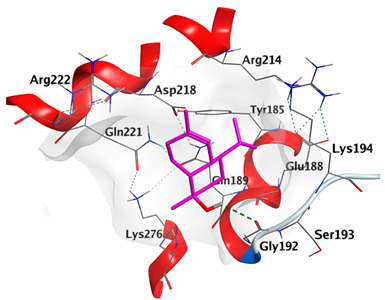
*δ*-Muurolene	−4.4583	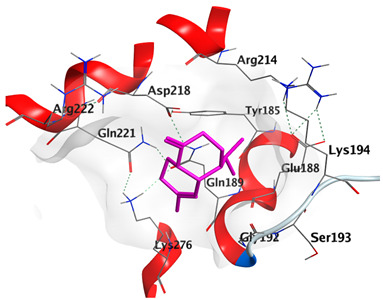
*α*-Pinene	−4.1318	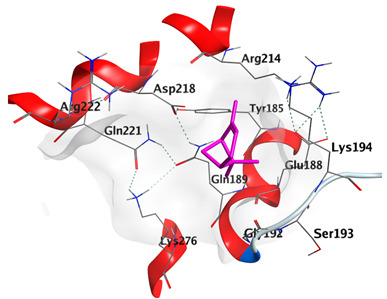

**Table 6:** Docking details of the selected components of *Chamaecyparis lawsoniana* leaf essential oil on caspase-8.

Component	S Score Kcal/mol	3D Protein-Component Interactions
*cis*-Abienol	−5.3517	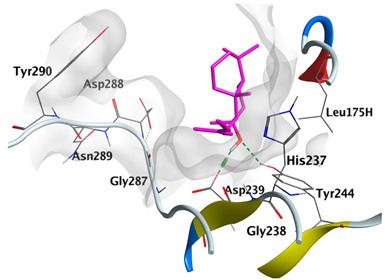
*trans*-Ferruginol	−5.4981	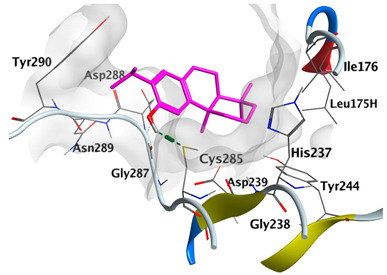
*α*-Cadinol	−5.0823	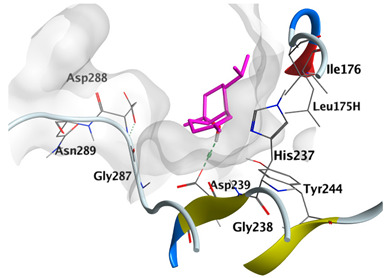
*δ*-Muurolene	−4.7582	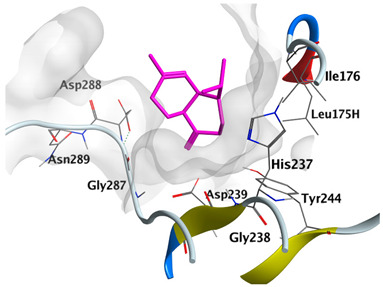
*α*-Pinene	−4.2315	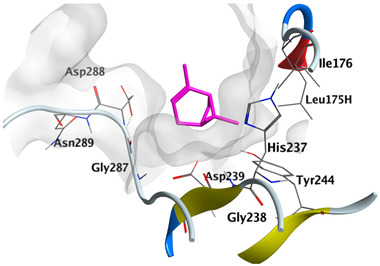

## Data Availability

All data and materials used are available in the manuscript.
